# HYpofractionated, dose-redistributed RAdiotherapy with protons and photons to combat radiation-induced immunosuppression in head and neck squamous cell carcinoma: study protocol of the phase I HYDRA trial

**DOI:** 10.1186/s12885-023-11031-w

**Published:** 2023-06-13

**Authors:** Joris B. W. Elbers, Pascal A. Gunsch, Reno Debets, Stijn Keereweer, Esther van Meerten, Jaap Zindler, Yvette van Norden, Mischa S. Hoogeman, Gerda M. Verduijn, Michiel Kroesen, Remi A. Nout

**Affiliations:** 1 Department of Radiotherapy, Erasmus MC Cancer Institute, Rotterdam, The Netherlands; 2 Department of Radiotherapy, HollandPTC, Delft, The Netherlands; 3 Department of Medical Oncology, Laboratory of Tumor Immunology, Erasmus MC Cancer Institute, Rotterdam, The Netherlands; 4 Department of Otorhinolaryngology head and neck surgery, Erasmus MC Cancer Institute, Rotterdam, The Netherlands; 5 Department of Medical Oncology, Erasmus MC Cancer Institute, Rotterdam, The Netherlands; 6 Department of Radiotherapy, Haaglanden Medical Center, Den Haag, The Netherlands

**Keywords:** Hypofractionation, Radiotherapy, HNSCC, Proton therapy, Immune system, Immunotherapy, lymphopenia, Elective nodal irradiation, Immunosuppression

## Abstract

**Background:**

Radiotherapy (RT) is the standard of care for most advanced head and neck squamous cell carcinoma (HNSCC) and results in an unfavorable 5-year overall survival of 40%. Despite strong biological rationale, combining RT with immune checkpoint inhibitors does not result in a survival benefit. Our hypothesis is that the combination of these individually effective treatments fails because of radiation-induced immunosuppression and lymphodepletion. By integrating modern radiobiology and innovative radiotherapy concepts, the patient’s immune system could be maximally retained by (1) increasing the dose per fraction so that the total dose and number of fractions can be reduced (HYpofractionation), (2) redistributing the radiation dose towards a higher peak dose within the tumor center and a lowered elective lymphatic field dose (Dose-redistribution), and (3) using RAdiotherapy with protons instead of photons (HYDRA).

**Methods:**

The primary aim of this multicenter study is to determine the safety of HYDRA proton- and photon radiotherapy by conducting two parallel phase I trials. Both HYDRA arms are randomized with the standard of care for longitudinal immune profiling. There will be a specific focus on actionable immune targets and their temporal patterns that can be tested in future hypofractionated immunoradiotherapy trials. The HYDRA dose prescriptions (in 20 fractions) are 40 Gy elective dose and 55 Gy simultaneous integrated boost on the clinical target volume with a 59 Gy focal boost on the tumor center. A total of 100 patients (25 per treatment group) will be recruited, and the final analysis will be performed one year after the last patient has been included.

**Discussion:**

In the context of HNSCC, hypofractionation has historically only been reserved for small tumors out of fear for late normal tissue toxicity. To date, hypofractionated radiotherapy may also be safe for larger tumors, as both the radiation dose and volume can be reduced by the combination of advanced imaging for better target definition, novel accelerated repopulation models and high-precision radiation treatment planning and dose delivery. HYDRA’s expected immune-sparing effect may lead to improved outcomes by allowing for future effective combination treatment with immunotherapy.

**Trial registration:**

The trial is registered at ClinicalTrials.gov; NCT05364411 (registered on May 6th, 2022).

## Background

Definitive radiotherapy with or without concurrent chemotherapy is standard of care (SOC) for most locally advanced head and neck squamous cell carcinoma (HNSCC). As the five-year overall survival is only 40% [1], there is an urgent need for additional solutions to improve clinical outcomes. PD-(L)1 immune checkpoint inhibitors provide an attractive combinatorial treatment for increasing survival in metastatic HNSCC with potential synergetic effects with radiotherapy as a radiosensitizer [2–4], However, unfortunately, it was recently reported that the combination of anti-PD-L1 avelumab with chemoradiotherapy did not result in a survival benefit [5].

Our hypothesis is that the combination of these individually effective treatments failed because of both radiation-induced immunosuppression and lymphodepletion. While subablative doses of radiotherapy are believed to be highly immunogenic [6], there are valid concerns regarding the immunosuppressive effect of fractionated, wide (elective) field radiotherapy [7–9]. First, adequate naive T cell priming by dendritic cell (DC) mediated MHC-I cross-presentation may be abrogated in defective draining lymph nodes after elective nodal irradiation (ENI) [10], resulting in a reduced number of intratumoral antigen specific CD8^+^ effector T cells [11]. Second, large radiation fields are extremely toxic for circulating lymphocytes, which are very sensitive to radiotherapy [12]. Therefore, we believe that the key lies in reforming conventional fractionated radiotherapy, which typically consists of large radiation fields in 35 fractions.

For the improvement of clinical outcome in HNSCC through immunotherapy and radiotherapy combinations, HYpofractionated Dose-redistributed RAdiotherapy (HYDRA) offers a promising strategy. First, the elective lymphatic field dose can be reduced by the combination of advanced multimodality imaging [13]. Second, radiation volumes can be reduced without impairment of local control by state-of-the-art image-guided radiotherapy and dose delivery [14]. With these radiotherapy advancements, it has also become technologically feasible to redistribute the radiation dose towards a lower dose on the outside of the target volume, while increasing the dose per fraction inside the tumor [15]. Therefore, the total number of fractions can be reduced (hypofractionation) which may result in less lymphocyte damage. Furthermore, by using protons instead of photon radiotherapy the integral radiation dose can be lowered, additionally resulting in immune cell damage reduction [16]. Apart from the possible immune-sparing effect of HYDRA, recent insights in modern radiobiology suggest that hypofractionated radiotherapy may result in higher tumor control with less radiation-induced toxicity, by more efficiently targeting accelerated repopulation [17, 18], and/or because the alpha/beta of HNSCC may be lower than previously assumed [19].

The objectives of this study are to determine the safety of HYDRA given by proton or photon radiotherapy by conducting two parallel phase-I trials. HYDRA’s efficacy will be compared to the standard of care. The immune effects of HYDRA-protons will be evaluated by longitudinal immune profiling and compared to those of HYDRA-photons and SOC (with protons and photons). There will be a specific focus on actionable immune targets and their temporal patterns that can be tested in future hypofractionated immune-radiotherapy trials.

## Methods

**Fig. 1:**
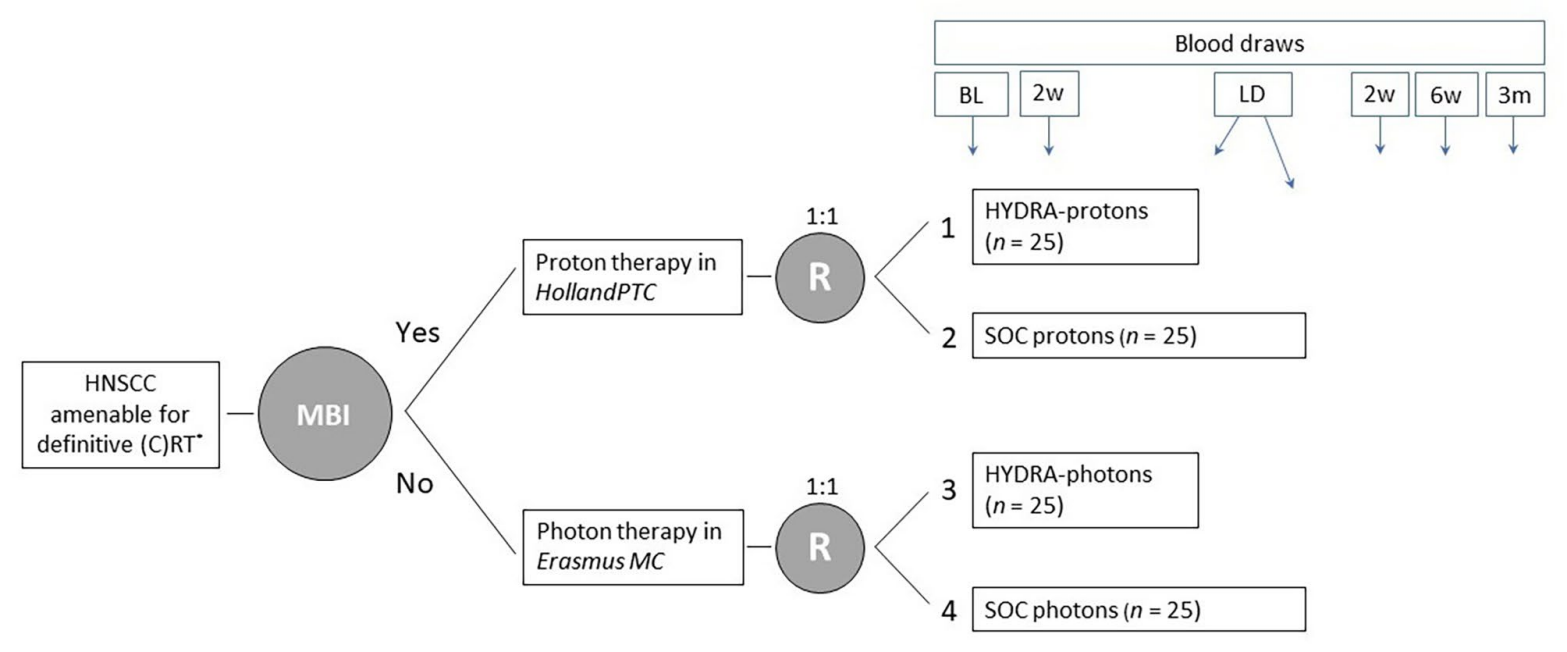
HYDRA study design: two parallel, noncomparative, open label phase-I trials. Randomization is performed for translational research purposes. ^*^ Radiotherapy with/without concurrent radiosensitizer. Oropharyngeal and hypopharyngeal carcinoma are amenable for inclusion. Laryngeal carcinomas are initially excluded until these patients are also considered eligible after interim analysis. Abbreviations: BL: baseline. LD: last day of treatment. SOC: standard of care. HNSCC: head and neck squamous cell carcinoma. MBI: model-based indication for proton therapy, according to the Dutch model-based selection criteria. R: randomization by minimization based on HPV status (positive vs. negative), stage (I-II vs. III-IV), and concurrent radiosensitization (yes vs. no)

A general overview of the treatment and study groups 1 to 4 is shown in Fig. 1, and described in more detail in the “design” section.

### Primary aim

To assess safety in terms of late grade 3–4 toxicity one year after the last patient has completed HYDRA for 25 HNSCC patients treated with HYDRA with proton therapy at HollandPTC (group 1) and in 25 HNSCC patients treated with HYDRA photon radiotherapy at the Erasmus MC (group 3). Patients will subsequently receive follow-up according to the standard of care up to five years after treatment for the evaluation of very late-onset toxicity. HYDRA is randomized with SOC radiotherapy for translational research purposes; a direct comparison of toxicity will statistically not be conclusive and is outside the scope of this study.

### Secondary aims

- To evaluate the objective response rate three months after HYDRA (groups 1 and 3), defined by radiological response on CT or MRI (in combination with an FDG-PET scan for node positive disease) and/or histopathological confirmation of residual disease, in comparison to groups 2 and 4, respectively.
- To determine the efficacy of HYDRA (groups 1 and 3) in terms of in-field and nodal elective field tumor control, 1 year after the last patient is included, in comparison to group 2 and 4, respectively.
- To correlate the numbers of immune cell populations and the frequency of T cell subsets according to markers of maturation, activation, cosignaling, and chemoattractant receptors at baseline, with patient- and tumor parameters (tumor localization, TNM status, tumor subtype, comorbidities etc.) in groups 1–4.
- To monitor temporal changes in immune markers obtained in blood during/after treatment at six timepoints as specified in Fig. 1, and differences in these changes between groups 1–4.

### Design

The safety of HYDRA will be evaluated in two parallel, noncomparative open label phase-I trials of patients treated with HYDRA-proton therapy (group 1) at HollandPTC and HYDRA-photon therapy (group 3) at Erasmus MC, The Netherlands. Patients will be selected for proton therapy according to standard of care Dutch model-based criteria. In brief, the protocol consists of a comparison of the photon plan and proton plan for the most favorable values of normal tissue complication probabilities (NTCPs) for dysphagia and xerostomia (grade ≥ II) [20].

For translational research purposes, each of the two parallel phase I trial cohorts will be compared with a study group receiving the standard of care treatment. To that end, HYDRA-proton therapy (group 1, *n* = 25) will be randomized by minimization in a 1:1 ratio with conventional fractionated proton therapy (group 2, *n* = 25), and HYDRA-photon radiotherapy (group 3, *n* = 25) will be randomized with conventional fractionated photon radiotherapy (group 4, *n* = 25). Randomization will be computer-generated, and minimization factors for randomization are: HPV status (positive vs. negative), tumor stage (I–II vs. III–IV) and the use of a concurrent radiosensitizer (yes vs. no). The PD-L1 Combined Positive Score (CPS) will be retrospectively determined and cannot be used for stratification, as the assessment typically takes multiple weeks and is not part of the initial work up.

We will determine efficacy to gain insight into whether HYDRA holds potential as a clinically effective hypofractionation protocol that should be tested in a subsequent trial. To increase statistical power, and because proton therapy is considered iso-effective to photon therapy, the efficacy of HYDRA will be determined for groups 1 and 3 combined and compared to the efficacy of conventional fractionated radiotherapy (groups 2 and 4). HYDRA should at least be iso-effective to conventional fractionated radiotherapy.

### Study participants

Patients with histology-proven squamous cell carcinoma of the oropharynx and hypopharynx, who are eligible for curative intent proton or photon therapy (with or without concurrent radiosensitizer) are amenable for inclusion. Patients must be 18 years or older and have WHO performance status of 0–2. Patients with laryngeal carcinoma are initially excluded until these patients are also considered eligible for treatment with HYDRA as described in the safety and statistics section. The main exclusion criteria are previous irradiation, chronic inflammatory disease or immune disorder and other malignant disease within the last two years.

### Interventions

#### HYDRA

The HYDRA dose prescriptions are delivered in 20 fractions. Gross tumor volume (GTV) is determined by the combination of PET-CT/MRI and ultrasound. The prescriptions consist of a focal inhomogeneous boost to the GTV of both the primary tumor and the pathological lymph nodes (CTV_5900 = GTV − 3 mm), a “conventional” simultaneous integrated boost (SIB) on the clinical target volume (CTV) of 55 Gy (CTV_5500 = GTV + 5 mm) and elective field radiotherapy of 40 Gy (CTV_4000 = GTV + 10 mm + elective lymph nodes). Delineations are in accordance with Grégoire et al. [21] and Biau et al. [22]. For the 55Gy and 40Gy CTVs, a 3 mm setup and 3%-range robustness is applied in case the patient is treated with proton therapy or a planning target volume (PTV) expansion margin of 3 mm is applied for photon radiotherapy. Target coverage is evaluated according to standard of care (D98 > 94% in voxel-wise minimum plan for protons, and D98 > 95% of the PTVs for photons [23]).

The focal boost to the GTV is delivered with a mean dose of 59 Gy and a maximum dose of 63.13 Gy (107% of 59 Gy). No more than 2% of the GTV should receive more than 63.13 Gy. The mean dose of 59 Gy corresponds to an equal late normal tissue toxicity probability after conventionally fractionated radiotherapy of 70 Gy in 35 fractions, considering an α/β = 3 for normal tissue. A 3 mm contraction margin is created to ensure that the 59 Gy boost always lies within the GTV (analogous to a negative PTV). If, after this step, there is no GTV_5900 volume left in the case of small tumor bulk, only a CTV_5500 boost will be prescribed. The mean dose of 59 Gy is a soft constraint: it is preferable but not obligatory and may be lower, depending on the 55 Gy boost dose constraints to the CTV.

The boost on the CTV has a mean dose of 55 Gy with an inhomogeneous distribution between 52.5 Gy at the border (95% of 55 Gy) and a maximum dose of 63.13 Gy within the GTV (107% of 59 Gy). No more than 2% of the CTV_5500 minus the GTV_5900 should receive more than 58.85 Gy (107% of 55 Gy). The volume that receives > 58.85 Gy should ideally be within the GTV_5900, but it is mandatory that this does not lie outside the CTV_5500. Discontinuation and treatment modification strategies are in accordance with the standard of care.

#### Standard of care (SOC)

Standard of care dose prescriptions consist of an elective dose of 54.25 Gy and a simultaneous integrated boost of 70 Gy to the primary tumor and pathologic lymph nodes in 35 fractions, five or six fractions per week.

#### Concomitant systemic therapy

Patients who receive HYDRA, as well as patients who receive standard of care treatment, may require the addition of a concurrent radiosensitizer based on clinicopathological features according to the standard of care.

### Safety, interim analysis and statistics

While there are reports about acceptable acute toxicity following hypofractionated chemoradiotherapy in advanced stage HNSCC [24–26], concerns about late toxicity remain, especially for laryngeal carcinoma. Therefore, an independent Data Safety Monitoring Board (DSMB) is established to perform ongoing safety surveillance and planned interim analyses for HYDRA-protons and HYDRA-photons independently.

After the tenth included patient has reached six months of follow-up, the DSMB will be consulted on whether to terminate the study, continue or expand the inclusion criteria with tumors originating from the larynx. Toxicity after six months is considered a preliminary surrogate for late-onset toxicity. Accrual will not be interrupted until the tenth patient has reached six months of follow-up, so at the time of interim analysis we expect that approximately 15 patients per treatment arm will be included, under the assumption that accrual will be linear. However, only the first ten patients with a follow-up of 6 months will be evaluated.

This decision for trial continuation is based on the number of patients who experience dose limiting toxicities (DLTs). A DLT is defined as grade 3–4 toxicity > 6 months after radiotherapy. Grade 3–4 toxicity should be considered causally related to radiotherapy, i.e. in addition to symptoms/increased severity than already present at baseline. The prevalence of DLTs is conventionally determined to be 33% for phase-I trials with a recommended acceptable dose for phase-II just below this toxic dose level [27]. This is in line with data derived from a phase-III trial evaluating standard of care chemoradiotherapy for oropharyngeal carcinoma [28]. At six months follow-up (− 6 to + 8 weeks), 13% of patients had grade 3–4 toxicity, and 17% was tube-feeding dependent [9]. Therefore, an additional expansion cohort of ten patients can be included (up to a total number of 25 included patients in total per treatment arm) if 3 or fewer patients experience grade 3–4 toxicity at six months after radiotherapy. If up to one patient experiences grade 3–4 toxicity after six months, laryngeal carcinoma patients may also be included. If more than three patients have grade 3–4 toxicity, the study must be prematurely ended.

In addition to the interim analysis criteria, the trial will be terminated prematurely in case one of the following stopping rules: one patient with radiotherapy-related death; more than two patients with radiotherapy-related grade 4 toxicity (i.e. an indication for operative intervention because of osteoradionecrosis, ulcer/fistula or bleeding); or more than four patients with radiotherapy-related tube-feeding dependency for more than six months.

For definitive conclusions regarding safety, late toxicity will be determined one year after the last patient has completed HYDRA. This will result in a median follow-up of approximately two years. In the phase-III trial by Gillison et al., there were 10% late grade 3–4 toxicities at one year after treatment [28]. At the final analysis, in case there are five patients (i.e. ≥20% of 25 included patients) experiencing grade 3–4 late toxicities after one year, HYDRA will be considered not feasible.

### Sample size

The stepwise cohort expansion as described above will result in 25 patients per treatment arm. As this is a phase-I trial evaluating HYDRA for multiple tumor subsites and stages with limited patients per treatment group, we anticipate significant heterogeneity. Although this is generally acceptable for a phase-I trial, it should be considered when comparing the immune effects of HYDRA with the standard of care. The immune profile analyses (according to earlier publications [29–31]) in this trial are exploratory in nature and the effect size in this treatment setting is currently unknown. Pragmatically, a number of 25 patients per treatment group is considered a good balance between (1) adequate evaluation of late toxicity, (2) limiting the number of patients at risk and (3) enough patients for exploratory immunological comparisons between treatment cohorts.

## Discussion

In the context of HNSCC, hypofractionation has historically only been reserved for small tumors out of fear for late normal tissue toxicity. The prevailing radiotherapy dose prescriptions were developed in the early 1950s and have hardly changed ever since, despite major technological advancements and modern radiobiology insights.

First, the combination of advanced radiologic imaging (ultrasound, CT, MRI, PET-CT) has led to a “transformation of the target volume”, which means that the occult tumor volume within the elective field has decreased, suggesting that a 36 Gy equivalent dose in 2 Gy fractions (EQD2) is nowadays sufficient to eradicate occult tumors [13]. Indeed, unpublished preliminary data from a Dutch multicenter randomized phase-III trial (UPGRADE, NCT02442375) show only three regional recurrences in both arms after 210 included patients and two years of follow-up (van den Bosch & Kaanders et al., presented at the annual Dutch NWHHT meeting, 2022). In addition, a group of Belgium colleagues has shown that 20 × 2 Gy elective field radiotherapy only results in 3.9% elective field recurrence at two years in 233 patients [32], which is comparable to the standard of care [33].

Second, accelerated repopulation of tumor cells is a well-known aspect within fractionated radiotherapy, and is historically assumed to start at a fixed time, with repopulation rates independent of the number of clonogens killed. However, a group from Columbia University has postulated that the onset time and rate of accelerated repopulation depend on the number of clonogens killed, and thus on dose and dose fractionation [17]. The alternative dose-dependent model (tested on data from 16 randomized HNSCC trials; 7283 patients) suggests that hypofractionated radiotherapy by 18 × 3 Gy (which is biologically equivalent to the proposed 20 × 2.75 Gy boost dose in our study) would result in improved tumor control, with less late toxicity [18].

Third, both intensity-modulated and image-guided radiotherapy (IMRT and IGRT, respectively) have been the standard of care for almost twenty years now, and have led to significant irradiation volume reduction without compromising clinical outcome [14]. Analysis of IMRT treatment reveals that recurrences are predominantly within the high dose target areas [34]. Optimizing local control without increasing toxicity can thus be achieved by redistributing the radiation dose, creating an inhomogeneous dose distribution towards the most (FDG-avid) active part of the tumor, instead of a conventional homogenous dose distribution with a 1 cm margin around the tumor area. This concept has formed the basis for the ARTFORCE trial, for which data are to be expected within the coming year [15]. As the dose directly outside the tumor is not increased, tumor control may be improved without additional toxicity. This concept of dose redistribution has already been proven in a randomized phase-III trial for prostate cancer [35].

A group from Brazil performed a phase-I trial of 20 × 2.75 Gy hypofractionated radiotherapy with concurrent cisplatin in advanced stage (75% stage IV) HNSCC and found that the acute toxicity rate was comparable to that of standard radiotherapy with concomitant chemotherapy [26]. Another prospective trial by the Mehanna group also showed that 25 × 2.6 Gy with concurrent chemotherapy resulted in tolerable acute toxicity in locally advanced oropharyngeal carcinoma [24]. From this, we may conclude that hypofractionation for HNSCC is well tolerated, at least in the acute phase and in large volume tumors, with and without the use of a concurrent radiosensitizer. Recently, a subgroup analysis of the PET NECK study was reported to support the use of a hypofractionated regimen during the COVID pandemic [25]. In this trial, 564 patients with locally advanced HNSCC receiving chemoradiotherapy were randomized to a planned neck dissection or active surveillance by FDG-PET. Three radiotherapy fractionation schedules (7, 6 or 4 weeks) were permitted: 56 patients received 20 × 2.75 Gy. There were no significant differences between the three fractionation schemes in terms of locoregional control, overall survival and quality of life after a minimum follow-up of two years.

We performed a planning study of HYDRA dose prescriptions in comparison to conventional fractionation for cT1-4N0-3M0 oropharyngeal (*n* = 4) and hypopharyngeal (*n* = 5) HNSCC patients. For all nine patients, hypofractionated, dose-redistributed radiotherapy allowed for an inhomogeneous boost up to 63 Gy within the macroscopic tumor and a dose reduction in all surrounding organs at risk (OAR) in comparison to standard of care radiotherapy. These results were presented at ECHNO-ICHNO 2021 [36].

With HYDRA, the overall treatment time is four weeks instead of the conventional 6–7 weeks. Patients will therefore receive only four courses of weekly 40 mg/m² cisplatin (160 mg/m² cumulative dose). There are data indicating that a minimal total dose of 200 mg/m² per treatment is associated with improved locoregional control [37]. However, in this setting the cisplatin dose does not lead to a reduction in distant metastasis [38]. From this, we conclude that a minimum dose of 200 mg/m² is not mandatory as a fixed threshold, but can be considered a surrogate of the number of weeks that a patient has received adequate radiosensitization (i.e. at least 5 of 7 weeks in total). We therefore do not consider it necessary to increase the weekly dose of cisplatin in the HYDRA arm, which otherwise may also result in additional toxicity.

By conducting two parallel phase-I trials, it is the aim to prove that HYDRA dose prescriptions are safe, for both proton and photon radiotherapy and for larger tumors. As all HNSCC patients who apply for definitive therapy will directly benefit from a reduced overall treatment time of four weeks instead of seven weeks, this will result in a significant reduction in treatment burden and costs. Actually, the radiobiology models used for this trial and lowered radiation dose prescriptions predict improved tumor control against a reduction of side effects. In the case of favorable safety and efficacy results, this study can subsequently be expanded into a randomized efficacy trial with the ultimate goal of facilitating the clinical implementation of HYDRA as a standard of care. Fewer fractions also imply lower costs, which is highly attractive for the sustainability of healthcare systems, specifically regarding the reimbursement of proton therapy. By longitudinal extensive translational immune monitoring, the objective is to prove that the immune system will be significantly spared by HYDRA. Furthermore, patient- and tumor-specific parameters that contribute to radiation-induced lymphodepletion will be identified. The information gained by these analyses is essential to improve individualized treatment. Actionable immune targets and their temporal patterns will be identified, so they can be tested in future hypofractionated immunotherapy combination trials. In conclusion, this phase I trial is therefore a very important step towards future personalized immune-radiotherapy combinations with the ultimate goal of improving survival for patients with HNSCC.

## Data Availability

Not applicable.

## Abbreviations

CPS: Combined positive score
CTCAE: Common Terminology Criteria for Adverse Events
CTV: clinical target volume
DC: dendritic cel
DLT: dose limiting toxicity
DSMB: Data Safety Monitoring Board
ENI: elective nodal irradiation
GTV: gross tumor volume
HNSCC: head and neck squamous cell carcinoma
HPV: human papillomavirus
HYDRA: HYpofractionated Dose-redistributed RAdiotherapy
IGRT: image guided radiation therapy
IMRT: intensity modulated radiation therapy
MBI: model-based indication
MHC: major histocompatibility complex
NTCP: normal tissue complication probability
OAR: organ at risk
OS: overall survival
PD-L1: programmed death-ligand 1
PFS: progression free survival
PROM: patient-reported outcome measure
PTV: planning target volume
SIB: simultaneous integrated boost
SOC: standard of care
WHO: World Health Organization
